# The Emerging Role of SGK1 (Serum- and Glucocorticoid-Regulated Kinase 1) in Major Depressive Disorder: Hypothesis and Mechanisms

**DOI:** 10.3389/fgene.2020.00826

**Published:** 2020-08-05

**Authors:** Vincenzo Dattilo, Rosario Amato, Nicola Perrotti, Massimo Gennarelli

**Affiliations:** ^1^Genetic Unit, IRCCS Centro San Giovanni di Dio Fatebenefratelli, Brescia, Italy; ^2^Department of Health Sciences, Magna Graecia University of Catanzaro, Catanzaro, Italy; ^3^Medical Genetics Unit, Mater Domini University Hospital, Catanzaro, Italy; ^4^Department of Molecular and Translational Medicine, University of Brescia, Brescia, Italy

**Keywords:** major depressive disorder, SGK1, neurodevelopment, stress, inflammation, neurotrophins, neurogenesis, antidepressant

## Abstract

Major depressive disorder (MDD) is a heterogeneous psychiatric disease characterized by persistent low mood, diminished interests, and impaired cognitive and social functions. The multifactorial etiology of MDD is still largely unknown because of the complex genetic and environmental interactions involved. Therefore, no established mechanism can explain all the aspects of the disease. In this light, an extensive research about the pathophysiology of MDD has been carried out. Several pathogenic hypotheses, such as monoamines deficiency and neurobiological alterations in the stress-responsive system, including the hypothalamic–pituitary–adrenal (HPA) axis and the immune system, have been proposed for MDD. Over time, remarkable studies, mainly on preclinical rodent models, linked the serum- and glucocorticoid-regulated kinase 1 (SGK1) to the main features of MDD. SGK1 is a serine/threonine kinase belonging to the AGK Kinase family. SGK1 is ubiquitously expressed, which plays a pivotal role in the hormonal regulation of several ion channels, carriers, pumps, and transcription factors or regulators. SGK1 expression is modulated by cell stress and hormones, including gluco- and mineralocorticoids. Compelling evidence suggests that increased SGK1 expression or function is related to the pathogenic stress hypothesis of major depression. Therefore, the first part of the present review highlights the putative role of SGK1 as a critical mediator in the dysregulation of the HPA axis, observed under chronic stress conditions, and its controversial role in the neuroinflammation as well. The second part depicts the negative regulation exerted by SGK1 in the expression of both the brain-derived neurotrophic factor (BDNF) and the vascular endothelial growth factor (VEGF), resulting in an anti-neurogenic activity. Finally, the review focuses on the antidepressant-like effects of anti-oxidative nutraceuticals in several preclinical model of depression, resulting from the restoration of the physiological expression and/or activity of SGK1, which leads to an increase in neurogenesis. In summary, the purpose of this review is a systematic analysis of literature depicting SGK1 as molecular junction of the complex mechanisms underlying the MDD in an effort to suggest the kinase as a potential biomarker and strategic target in modern molecular antidepressant therapy.

## Introduction

Major depressive disorder (MDD) is the most common psychiatric illness and a global public health problem (World Health Organization [WHO], 2020a). MDD is a symptomatically heterogeneous disease characterized by prominent and persistent low mood, loss of interest, low self-esteem, cognitive impairment, volitional decline, and vegetative symptoms, such as disturbed sleep or appetite. Due to these clinical symptoms and the high recurrence rate, MDD is the third leading cause of years lived with disability worldwide and a major contributor to suicide risk (James et al., 2018). It is estimated that about 50% of the 800,000 suicides per year worldwide occur among subjects with MDD, which presents a 20-fold more risk of dying by suicide compared to the general population (World Health Organization [WHO], 2020b; Chesney et al., 2014).

Parental and twin-based studies estimated a 37% chance of heritability, thus suggesting a genetic contribution to MDD (Flint and Kendler, 2014). Candidate gene association studies, widely used over the past 40 years in the genetic analysis of MDD, analyzed more the 100 candidate genes in order to identify the possible associations between their alleles and the risk of depression occurrence. This approach uses genes selected *a priori* based on their biological function and involvement in neurobiological mechanisms underlying MDD (Shadrina et al., 2018). Despite candidate gene association studies on MDD have revealed several suspected risk genes, the results obtained are conflicting. Indeed, even though a clear biological function of the gene may exist, this information may not always be complete, leading to incorrect assumptions about these functions and therefore not always reflecting in an associated outcome. Moreover, these studies do not consider indirect associations as the interactions between gene and environment (Verbeek et al., 2014). The advent of genome-wide genotyping techniques with DNA microchip technology gave the opportunity to conduct genome-wide associations studies (GWASs) with the aim to identify risk factors of depression onset independently from the starting hypotheses and using large sets of samples. However, since MDD is a complex disease arising from the combined effect of many small-size genetic variants, GWAS, identifying single nucleotide polymorphisms (SNPs) across the genome transmitted in linkage disequilibrium with a causative polymorphism, have had notable difficulties in identifying individual associated loci and in replicating significant findings (Bosker et al., 2011). More recent studies have proven moderately successful in the identification of several risk variants significantly associated with MDD, mainly located in genes involved in neurodevelopmental, inflammatory, and oxidative stress processes (Cai N. et al., 2015; Hyde et al., 2016; Okbay et al., 2016; Wray et al., 2018; Coleman et al., 2019; Howard et al., 2019). However, larger sample sizes or genetic isolates are needed to robustly detect specific risk loci given their effect sizes, thus overcoming the population heterogeneity. Furthermore, environmental factors, mainly sexual, physical, or emotional trauma during childhood, are strongly associated with an higher risk (from 2.66 to 3.73 times) of MDD occurrence in adulthood (Li et al., 2016; Nelson et al., 2017). Despite advances in the understanding of MDD pathophysiology, the causative mechanisms underpinning the interaction of environmental, genetic, and epigenetic factors are still far from being clarified. To date, several pathogenetic hypotheses of MDD have been proposed, centering around monoaminergic systems, hypothalamic-pituitary-adrenal (HPA) axis dysregulation, inflammatory/immunological dysfunction, neuroplasticity, or neurogenesis alterations. Although they have been described as distinct pathogenetic hypotheses, they effectively appear to be linked in inducing the pathological phenotype. Haase and Brown (2015) reviewed a large body of published findings and proposed a model in which serotoninergic transmission and neurotrophins signaling are reciprocal interconnected in condition of inflammation-induced depression.

The monoamine-deficiency hypothesis was the first theory that has been proposed about the molecular mechanisms underlying MDD. Many of the antidepressant drugs that are currently used in the treatment of MDD exert their effects by increasing the availability of the monoamine neurotransmitters serotonin (or 5-hydroxytryptamine, 5-HT), noradrenaline (or norepinephrine, NE), and dopamine (DA) in the brain (Pitsillou et al., 2019). Although these drugs affect the neurotransmitter systems within hours after administration, the improvement of symptoms is often evident only after several weeks (4 to 6 weeks) of treatment, and this is a relevant problem in clinical practice (Liu B. et al., 2017).

The delayed efficacy of antidepressants can be explained by the neurotrophic hypothesis, a new molecular theory in the pathogenesis of MDD that does not rule out, but rather strengthens, the previous monoaminergic theory. The neurotrophic hypothesis arises from evidence that revealed an action of antidepressant drugs on neurotrophins, a class of small proteins supporting neural survival in embryonic development and promoting differentiation, enabling axonal growth, driving nerve-growth direction, preserving the survival of mature neurons, and accelerating neurogenesis (Levy et al., 2018). In particular, antidepressant drugs are known to enhance neuronal trophism by counteracting the reduction of axon growth and the abnormalities in dendritic arborization and spine density observed in animal models of depression. This improvement derives from the antidepressant-dependent increase in expression of genes encoding for the neural growth factor (NGF), and other neurotrophic factors, including the brain-derived neurotrophic factor (BDNF), the glial cell-derived neurotrophic factor (GDNF), and the vascular endothelial growth factor (VEGF) (Levy et al., 2018). However, the increase in neurotrophic factors proceeds hand-in-hand (some weeks) with the alleviation of symptoms under antidepressant treatment, thus suggesting that the onset of the pharmacological efficacy is not exclusively related to an increase in the level of monoamines, but may be the consequence of the restoration of a normal neuronal function, resulting from the action of neurotrophic factors (Neto et al., 2011). Moreover, the neurotrophic hypothesis is corroborated by the size restoration of different brain areas involved in controlling mood, such as hippocampus, prefrontal cortex, and nucleus accumbens, after long-term pharmacological treatment (Liu W. et al., 2017). However, post-mortem human studies are often inconsistent and conflicting with the neurogenic theory. Reif et al. (2006) revealed no differences in the proliferation of hippocampal neural stem cells in brain samples of depressed patients compared to control subjects. Moreover, the proliferation rate appeared to be not modified by antidepressant drug treatment (Reif et al., 2006). On the other hand, some studies showed a significant decrease in hippocampal progenitor cell number of untreated depressed subjects (Lucassen et al., 2010), an effect counteracted by treatments with selective serotonin-reuptake inhibitors (SSRIs) and tricyclic antidepressants (TCAs) (Boldrini et al., 2009; Lucassen et al., 2014). Taken together, these conflicting findings denote that the role of neurogenesis in human depression remains elusive, thereby undermining the veracity of the neurogenic theory.

Another theory about the pathogenesis of MDD is the stress hypothesis based on the hypothalamic-pituitary-adrenal (HPA) axis dysregulation. The HPA axis is an important component of neuroendocrine system consisting of three parts: the hypothalamic area, the pituitary gland, and the adrenal cortex. Briefly, in response to environmental stimuli, neurons of hypothalamic area synthesize and release corticotropin-releasing hormone (CRH), thus promoting synthesis and release of adrenocorticotropic hormone (ACTH) by the pituitary gland, which, in turn, induces the adrenal cortex to produce and to secrete glucocorticoids (mainly cortisol). The HPA axis hyperactivity is an evident clinical manifestation in patients with MDD, showing increased secretion of CRH, ACTH, and glucocorticoids in the cerebrospinal fluid as well as elevated blood levels of glucocorticoids (Black, 2002; Zunszain et al., 2011). High concentration of glucocorticoids can play long-term negative effects, including the malfunction of negative feedback along the HPA axis and the excessive activation of glucocorticoids receptor (GR) in central nervous system (CNS) target cells. The aberrant GR activation leads to neuronal apoptosis and degeneration (Kino, 2015) through the reduction of both BDNF expression and cell proliferation (Chen et al., 2017; Odaka et al., 2017). Moreover, high levels of glucocorticoids are also able to enhance the GR-dependent expression of 5-HT transporter in the hippocampus, frontal cortex, amygdala, and other brain regions, thus inducing a decrease of 5-HT in the synaptic cleft and a further exacerbation of depressive symptoms (Cai S. et al., 2015). However, only approximately 50% of depressed patients exhibit disturbances of the HPA system, thus denoting the high heterogenicity of MDD (Keller et al., 2017).

Increasing evidence from animal studies as well as clinical observations in depressed patients support a role for inflammation and immune dysfunction in the occurrence of MDD. The rationale behind the inflammatory/immune hypothesis derives from the involvement of immune systems in the physiological stress-sensing pathways and its interaction with HPA axis, autonomic nervous system (ANS), and central nervous system (CNS) by a mutual regulation. Animal models of depression revealed how the cytokines, once transported through the blood brain barrier by specific endothelial cell transporter or diffusing through deficient barrier areas, can directly or indirectly affect brain circuits, behaviors, and mood, acting on neurons, astrocytes, and microglia (Kennedy and Silver, 2016; Ambrée et al., 2018). Furthermore, inflammatory signals can converge on CNS through the vagus nerve or by means of infiltrating peripheral immune cells. Animal models also revealed that, regardless of the signaling involved, inflammatory stimuli were able to alter the cellular expression pattern in CNS, thus affecting neurogenesis and plasticity (Hodes et al., 2015). In humans, severe infections as well as autoimmune diseases were described to be closely related with higher risk to develop MDD (Benros et al., 2013). In addition, meta-analyses displayed increased blood expression of pro-inflammatory cytokines and macromolecules such as tumor necrosis factor alpha (TNF-α), interleukin 6 (IL-6), and C reactive protein (CRP) in patients with MDD (Dowlati et al., 2010; Haapakoski et al., 2015). In a large-scale cohort study, genes belonging to IL-6 signaling have also been described to be increased in peripheral blood cells of patients with MDD compared with healthy controls (Jansen et al., 2016). Moreover, prospective studies showed that increased serum levels of IL-6 during childhood significantly increased the risk to develop MDD and psychosis in young adulthood (Khandaker et al., 2014). An elevated expression of TNF-α, IL-6, and other proteins related to innate immunity, such as interleukin 1β (IL-1β) and toll-like receptors 3 (TLR3) and 4 (TLR4), has been observed also in the brain of patients with MDD (Pandey et al., 2014, 2018). High levels of IL-1β, IL-2, and TNF-α can induce neuronal apoptosis, inhibit neuronal differentiation, abolish synaptic transmission, and counteract either the induction or maintenance of long-term potentiation, thus leading to an impairment of learning and further worsening of depression symptoms (Cai S. et al., 2015). Inflammatory cytokines also cause glucocorticoid resistance and reduction in BDNF expression by blocking the functions of GRs, thus interfering with negative feedback in the HPA axis. This results in a reduction in BDNF brain levels that leads to apoptosis or degeneration of neurons (Jin et al., 2019; Perrin et al., 2019). Moreover, non-depressed patients, treated with cytokines such as IL-2 or interferon-γ (IFNγ) as a part of their therapy for hepatitis virus infection or cancer, developed depressive symptoms (Myint et al., 2009). Conversely, the pharmacological blockade of inflammatory processes by the selective cyclooxygenase 2 inhibitor celecoxib or TNF-α inhibitors had positive effects on depressive symptoms (Köhler et al., 2014; Abbott et al., 2015). Another interesting target is represented by the potassium channel Kv1.3. Its role in glial neuroinflammation and in the pathogenesis of nigrostriatal lesion of Parkinson’s disease, multiple sclerosis, and Alzheimer’s disease has recently been determined (Wang et al., 2020). To date, its role in the depressive spectrum complex is less clear. However, due to its excitatory role and being an indirect target of SGK1 (Lang and Shumilina, 2013), further experimental evaluations in animal models could provide interesting evaluations of its possible involvement as a diagnostic and therapeutic target in MDD. Finally, additional evidence corroborating the link between inflammation and depression are provided by neuroimaging and brain post-mortem studies that indicated neuroinflammation and microglial activation in the CNS of MDD patients (Enache et al., 2019; Liu et al., 2019).

A new player with diversified genetic and functional roles emerges to bind, like a molecular string, the various pathological and functional aspects within the intricate natural history of clinical manifestations, hidden below the definition of major depressive disorder (MDD): the serum- and glucocorticoid-regulated kinase 1 (SGK1).

Serum- and glucocorticoid-regulated kinase 1 is a serine/threonine kinase, part of the AGK Kinase family, that displays several homologies with AKT (Protein Kinase B), PKC (Protein Kinase C), and S6K (Ribosomal S6 Kinase) (Frödin et al., 2002; Mora et al., 2004; Bruhn et al., 2010). SGK1 is regulated via different mechanisms by insulin, cAMP (Perrotti et al., 2001; Boito et al., 2005; Menniti et al., 2005), IGF-1 (Insulin like growth factor-I) (Boini et al., 2008), steroids (Chen et al., 1999), IL-2 (Interleukin-2) (Amato et al., 2007), and TGFβ (Transforming growth factor-beta) (Lang and Voelkl, 2013) and has been depicted as a pivotal convergence point for peptide and steroid hormone regulation of ENaC-mediated Na^+^ transport (Faletti et al., 2002). Interestingly, several SGK1 polymorphic variants are associated with type 2 diabetes, obesity, and increased blood pressure in Caucasian and African populations (Schwab et al., 2008), whereas a strong link between heart disease and depression, both of which are closely related to lifetime stress exposure, has been verified in Chinese Han patients (Han et al., 2019).

In terms of downstream effectors, SGK1 controls ENaC (amiloride-sensitive sodium channel), other ion channels (e.g., KCNE1/KCNQ1), carriers (such as NCC, NHE3, SGLT1), Na(+)/K(+)-ATPase, enzymes (including glycogen-synthase-kinase-3), and transcription factors or regulators (including FOXO3a, β-catenin, NF-kappaB, SP1, p27) (Lang and Görlach, 2010; Schmid et al., 2014; Voelkl et al., 2015). Ion channel physiology and pathophysiology of membrane polarization appear to play an important role in cell proliferation, although rigorous scientific demonstrations are still lacking (Zhang et al., 2014; Zhou et al., 2015b). Taken together, all these information underline that SGK1, originally studied as a kinase responsible for regulating several cellular ion channels and pumps (Wulff et al., 2002; Lang et al., 2011; Chraïbi and Renauld, 2014), can indeed have a role in oncology and immunology as well (Lang et al., 2006; Sobiesiak et al., 2009; Wu C. et al., 2013). SGK1 function is directly dependent on mTOR phosphorylation. Following the mTOR-dependent hydrophobic motif (H-motif) phosphorylation on serine 422 (García-Martínez and Alessi, 2008), the kinase changes into an open conformation for phosphorylation and complete activation by 3-phosphoinositide-dependent kinase-1 (PDK1) (Hong et al., 2008). Copy number variation, as well as an increase in the expression and/or activity of SGK1, has been found in several human tumors (Abbruzzese et al., 2012; Sommer et al., 2013; Stringer-Reasor et al., 2015; Talarico et al., 2015, 2016a,b; Ma et al., 2019). Currently, SGK1 expression is described as related to events of invasiveness and metastasization (Huang et al., 2015; Qin et al., 2015; Xiaobo et al., 2016). Conversely, SGK1 knock-out models have been shown to be strongly resistant to chemical carcinogenesis (Nasir et al., 2009). It has recently been demonstrated that SGK1 is a crucial step in mediating cell survival, proliferation, and differentiation via phosphorylation of Mouse Double Minutes 2 (MDM2), which controls p53 ubiquitylation and proteasomal degradation (Amato et al., 2009). SGK1 also influences mitotic stability by affecting the expression of RANBP1 (Ran-specific binding protein 1), the pivotal regulator of GTPase RAN (Amato et al., 2013; D’Antona et al., 2019). More recently, it has been shown that SGK1 through RANBP1/RAN strictly regulates the nucleocytoplasmic transport of pre-miRNAs, a necessary condition for miRNAs maturation, thus modulating the epigenomic framework of the cell (Dattilo et al., 2017). This mechanism, which is so important in oncological diseases, can be of considerable interest also in psychiatric diseases such as major depression, in which neuronal epigenetic and epigenomic reorganization is widely discussed (Pitsillou et al., 2019). Interestingly, a new inhibitor for SGK1, named SI113, has been developed. In cellular models as well as in murine models, SI113 has amply demonstrated potential antineoplastic capacity, showing a very low toxicity profile (Abbruzzese et al., 2017, 2019; Catalogna et al., 2017; Matteoni et al., 2019). Pharmacological ADME (absorption, distribution, metabolism, and excretion) studies have corroborated the ability of SI113 to cross the blood-brain barrier, thus opening the possibility of a rational use of the molecule, even in psychiatric and neurological diseases (Talarico et al., 2016b). Interestingly, the most important molecular interactors as well as the downstream targets of SGK1 and its upstream modulators are involved in various ways in the etiopathogenesis of depression, constituting a clear link between this signaling pathway and the depressive pathogenesis (Table 1). It is also interesting to note how the whole FOXO3a system and the complex of modulating cytokines also open up a new etiopathogenetic hypothesis on an autoimmune Th17-dependent basis. In this light, the purpose of this review is precisely to outline, in the current knowledge framework, the interconnections between SGK1 and its modulations with the causative mechanisms of MDD, eventually clarifying the theoretical problems for the potential definition of SGK1 as a therapeutic target.

**TABLE 1:** Table illustrating the SGK1 signaling partners in the MDD.

Interaction/Upstream/Downstream effectors	Papers	References
RAN	Maternal Stress Predicts Altered Biogenesis and the Profile of Mitochondrial Proteins in the Frontal Cortex and Hippocampus of Adult Offspring Rats.	Głombik et al., 2015
NF-KappaB	BDNF/NF-κB Signaling in the Neurobiology of Depression.	Caviedes et al., 2017
SP1	Neurotrophic factor-α1 prevents stress-induced depression through enhancement of neurogenesis and is activated by rosiglitazone.	Cheng et al., 2015
mTOR	The role of mTOR in depression and antidepressant responses.	Abelaira et al., 2014
IL-2, TGFβ and cytokines	Comorbidity between depression and inflammatory bowel disease explained by immune-inflammatory, oxidative, and nitrosative stress; tryptophan catabolite; and gut-brain pathways.	Martin-Subero et al., 2016
FOXO3a	IGF-1 defends against chronic-stress induced depression in rat models of chronic unpredictable mild stress through the PI3K/Akt/FoxO3a pathway. Th17 cells in depression.	Beurel and Lowell, 2018; Kuang et al., 2018

*RAN, GTPase Ran; NF-KappaB, Nuclear Factor Kappa B; mTOR, mammalian Target of Rapamycin; IL-2, Interleukin 2; TGFβ, Transforming Growth Factor-beta; FOXO3a, Forkhead Box O3; BDNF, Brain-Derived Neurotrophic Factor; IFG-1, Insulin Like Growth Factor 1; PI3K, Phosphatidylinositol-4,5-Bisphosphate 3-Kinase; Akt, Protein Kinase B.*

## SGK1 and L-HPA Axis Activity

In humans, cortisol is the stress hormone that acts through the Glucocorticoid Receptor (GR) and the Mineralocorticoid Receptor (MR), both of which can act as transcription factors. Low blood levels of endogenous glucocorticoids bind MR with high affinity, enhancing proliferation of human hippocampal progenitor cells and differentiation into astrocytes. Conversely, increased levels of stress-induced glucocorticoids bind GR with low affinity, resulting in a decrease of the neural stem/progenitor cells (NSPCs) proliferation and neural differentiation (de Kloet et al., 1998; Anacker et al., 2013a). This evidence indicates that glucocorticoids can affect neurogenesis through a dual activity: a positive function (via MR) and a negative function (via GR) (Bose et al., 2010; Samarasinghe et al., 2011; Raciti et al., 2016). Severely depressed patients as well as rodent stress models are characterized by increased glucocorticoid levels (Anacker et al., 2011). Indeed, the most common biological feature in patients with MDD consist of chronically elevated glucocorticoid levels after a prolonged exposure to stress (Numakawa et al., 2013). SGK1, an ubiquitously expressed serine/threonine kinase, is transcriptionally regulated by a wide variety of factors, including glucocorticoids (Faletti et al., 2002). SGK1 is a protein involved in cellular stress response with a relevant role in neuronal function (Lang et al., 2010). In particular, SGK1 regulates the neuronal activity, proliferation, and apoptosis, becoming then a key determinant of susceptibility to mental illness (Han et al., 2019). The brain expression of SGK1 is positively regulated by glucocorticoids (Sato et al., 2008; Sarabdjitsingh et al., 2010), motor hyperstimulation (Kalinichev et al., 2008), and administration of the antipsychotic drug clozapine (Robbins et al., 2008). Hippocampal SGK1 expression is regulated by fear stimulation and after enrichment training (Lang et al., 2006). SGK1 transcript levels are also promoted by the psychostimulant amphetamine, hallucinogenic drug lysergic acid dimethylamide (LSD), and neuronal injury (Lang et al., 2006). Hypothalamic SGK1 levels are increased by fasting in conditions of obesity (Nonogaki et al., 2006). Experimental evidence points to a role of SGK1 in long-term memory formation (Ma et al., 2006). More recently has been demonstrated that SGK1 stimulates dendrite growth, thus probably impacting on learning abilities (Lang et al., 2006). Moreover, SGK1 also participates in the establishment of the spatial memory and neuronal plasticity through the phosphorylation of IKKα with subsequent stimulation of NFκB, thus leading to the expression of genes, such as *NR2A* and *NR2B* (Tai et al., 2009). In addition, SGK1 could affect neuronal excitation by modulating Kv channels (Lang et al., 2006, 2009), which, in turn, are essential and limiting in the preservation of neuronal membrane potential (Pongs, 2007). Interestingly, SGK1 has been proposed as repressor of the neurogenic Hedgehog pathway via GR. In human hippocampal progenitor cell line HPC03A/07, the GSK650394, a SGK1-small molecule inhibitor, counteracted the cortisol-induced suppression of Hedgehog signaling, resulting in proliferation and neuronal differentiation. Moreover, SGK1 was able to potentiate and maintain GR activation following cortisol stimulation by allowing GR phosphorylation at the serine residues S203 and S211, thus favoring its nuclear translocation. This implies that SGK1 is not only a downstream target of GR signaling, but also exerts a positive feedback on GR activation by regulating the long-lasting effects of glucocorticoids (Anacker et al., 2013b). Luca et al. recently identified a single-nucleotide polymorphism (rs9493857) lying in the *SGK1* promoter region within an Oct1 binding site, a transcription factor cooperating with GR in the transactivation of target genes. This polymorphism showed marked allele frequency between populations of African and European ancestry, allowing the definition of an ancestral and a derived allele. The ancestral allele, more prevalent in African populations, binds the GR-Oct1 complex more efficiently than the derived allele and is associated with increased glucocorticoid-dependent gene expression when compared with the derived allele (Luca et al., 2009). The role of SGK1 in impairing hippocampal neurogenesis is suggested by the evidence obtained on two rodent stress models characterized by depressive behavior and decreased hippocampal neurogenesis. In details, in rat models of depression induced by unpredictable chronic mild stress (UCMS) or early life stress, a significant increase of the hippocampal *SGK1* mRNA level was observed, together with a reduction in Hedgehog signaling, as expected (Anacker et al., 2013b; Bockmühl et al., 2015). A similar trend toward an increase in SGK1 gene expression was detected in peripheral blood of drug-free depressed patients (*n* = 25 vs. *n* = 14 healthy controls), thus confirming the evidence observed in rodents (Anacker et al., 2013b). Furthermore, increased levels of SGK1 were observed in the subgranular zone (SGZ) of the hippocampal dentate gyrus (DG) of *miR-17-92* KO mice, which exhibited reduced adult neurogenesis and anxiety- and depression-like behaviors. Otherwise, the direct targeting of SGK1 by miR-19a and miR-92a, belonging to the miR-17-92 cluster, over-expressed in *miR-17-92* over-expressing (OE) mice, resulted in the maintenance of proliferative neural progenitors and the generation of newborn neurons, producing anxiolytic and antidepression-like behaviors in these mice. The results indicate that miR-17-92 cluster deletion or overexpression in mice results in elevated anxiety-like or antianxiety-like behaviors, respectively (Jin et al., 2016). This finding suggests that the effects of miR-17-92 deletion in hippocampal neurogenesis and mouse behaviors are, at least in part, mediated by increased SGK1. A significant increase in *SGK1* mRNA was also observed, by means of next-generation RNA sequencing and pathway analysis, in the periaqueductal gray (PAG) of two mouse models of neuropathic pain [spared nerve injury (SNI)] and depression [chronic unpredictable stress (CUS)], revealing that SGK1 expression may indeed link, at a molecular level, pain and chronic stress (Descalzi et al., 2017). Interestingly, a recent paper showed that SGK3, an isoform highly expressed in the brain sharing 80% amino acid sequence identity with SGK1 in the catalytic domain (Kobayashi et al., 1999), induced SGK3-mediated autophagic cell death (ACD), but no apoptosis of hippocampal neural stem cells (NSCs), thereby determining decline of adult hippocampal neurogenesis and cognitive deficits (Jung et al., 2019). Although most of evidence points to the enhancement of SGK1 expression under chronic stress conditions, few papers describe a decreased or unaffected SGK1 expression after acute stress exposures. Licznerski et al. (2015) showed a significant reduction of *SGK1* mRNA in a small cohort of post-mortem Prefrontal Cortex (PFC) of patients diagnosed with Post Traumatic Stress Disorder (PTSD, *n* = 6) compared to age-matched individuals without psychiatric diagnosis (*n* = 6). The down-regulation of SGK1 replicated also at protein level in the PFC of a subgroup of rats undergone to inescapable stress paradigm. Furthermore, rats infused in the PFC with an adeno-associated virus carrying a dominant negative form of SGK1 (S422A) caused helplessness- and anhedonic-like behaviors (Licznerski et al., 2015). A very recent study on adult rats, subjected to acute stress during adolescence, revealed no significant changes in *SGK1* mRNA levels in anxiety-related brain regions such as central amygdala, medial amygdala, ventral hippocampus, and paraventricular nucleus (Lovelock and Deak, 2019). Despite the high comorbidity between MDD and PTSD, the modulation of SGK1 expression could be mediated by distinct signaling pathways. Indeed, the expression of genes strictly related to HPA axis, such *FKBP5* and *NR3C1* (gene encoding glucocorticoids receptor), appeared not influenced under these conditions, thereby suggesting that HPA axis regulation results unaffected by acute stress exposure. Recent evidence supports the idea that plasticity in adult brain white matter structure and myelination arises from the activity-dependent modulation of oligodendrocyte function (Gibson et al., 2014; Pepper et al., 2018) in response to various experiential events, including new training on cognitive task (Fields, 2015). Although the molecular mechanism behind this process is not well-known, SGK1 appears to be a potential mediator of oligodendrocyte plasticity due to its rapid upregulation in adult rat and mouse brain white matter after acute stress (Miyata et al., 2011). Indeed, a dynamic glucocorticoid-dependent regulation of SGK1 mRNA as well as its kinase activity induction were observed in oligodendrocytes of corpus callosum of acute stressed adult male rats (Hinds et al., 2017) and chronic stress exposed mice (Miyata et al., 2015), respectively. Active SGK1 led to N-myc downstream-regulated gene 1 (NDRG1) phosphorylation and, as downstream effect, to the increase of N-cadherin, α-catenin, and β-catenin expression, both *in vitro* and *in vivo*. The abundance in catenin/cadherins expression resulted, in turn, in morphological changes in the oligodendrocytes of corpus callosum nerve fiber bundles. Remarkably, the recovery from chronic stress restored both the arborization of oligodendrocytes and the depression-like symptoms, confirming that morphological changes of these cells are conceivably related to depressive behaviors (Miyata et al., 2011). Similarly, chronic stress induced the SGK1-dependent up-regulation of *Dsg1* encoding for the cell adhesion molecule named desmoglein 1, a calcium-dependent desmosomal cadherin. This finding could suggest that also Dsg1, a target of SGK1, may be involved in molecular mechanisms responsible for the oligodendrocyte morphological changes in response to chronic stress exposure (Miyata et al., 2015). Finally, the hypothalamic expression of *SGK1* was described to be increased in a mouse model of stress induced by social isolation. Injection of a small interfering RNA (siRNA) oligonucleotide, specific for *SGK1*, into the third cerebral ventricle blocked the acute social isolation-induced reduction in body weight and increase in plasma active ghrelin (a hunger stimulating hormone) levels (Kaji and Nonogaki, 2010). Taken together, these pieces of evidences strongly propose the involvement, with a key role, of SGK1 in the pathogenetic stress hypothesis of major depression (Table 2).

**TABLE 2:** Table illustrating the SGK1 involvement in humans, *in vitro* and *in vivo* models of stress.

Experimental model	Molecular features	Biological and clinical features	References
HPC03A/07 hippocampal progenitor cells exposed to cortisol	Hedgehog signaling suppression after SGK1 inhibition by GSK650394; Enhancing of GR nuclear translocation following its SGK1-dependent hyperphosphorylation at S203 and S211	Reduction in neural proliferation and differentiation	Anacker et al., 2013b
Drug-free depressed patients	Increased peripheral blood expression of SGK1	Depressive symptoms	Anacker et al., 2013b
Combination of population genetics	Increased GR/Oct1-dependent SGK1 expression in presence of rs9493857 polymorphism	Genetic variant associated with greater responsiveness of neuroendocrine signaling in response to environmental stressor stimuli, resulting in the predisposition of individuals to chronic disorder	Luca et al., 2009
UCMS-rats; Early life stressed rats	Increased hippocampal SGK1 mRNA levels; Reduction in Hedgehog signaling	Decreased hippocampal neurogenesis with depressive behaviors	Anacker et al., 2013b; Bockmühl et al., 2015
miR-17-92 KO mice	Increased SGK1 levels in the SGZ of hippocampal DG	Reduced adult neurogenesis with anxiety- and depressive-like behaviors	Jin et al., 2016
SNI and CUS mice	Increased SGK1 mRNA in the PAG	SGK1 as a linker between pain and chronic stress	Descalzi et al., 2017
Chronic stressed mice	SGK3-mediated autophagic cell death of hippocampal NSCs	Decline of hippocampal neurogenesis with cognitive deficits	Jung et al., 2019
Corpus callosum oligodendrocytes of chronic stress exposed mice	Increase in SGK1 mRNA and kinase activity; Increase in N-cadherin, α-catenin and β-catenin via SGK1/NDRG1 axis both *in vitro* and *in vivo*	Morphological changes in oligodendrocytes of corpus callosum nerve fiber bundles; Recovery from stress restored the normal arborization of oligodendrocytes as well as the depression-like symptoms	Miyata et al., 2011
Acute stressed adult rats	Increase in SGK1 mRNA in the corpus callosum	SGK1 as an important component of the interplay between oligodendrocytes and neuronal function including neuroplasticity	Hinds et al., 2017
Corpus callosum oligodendrocytes of chronic stress exposed mice; Primary oligodendrocytes exposed to dexamethasone	Increase in SGK1 expression/activity leading to Dsg1 up-regulation	Morphological changes in oligodendrocytes of corpus callosum	Miyata et al., 2015
Stressed mouse model by social isolation	Increase in hippocampal SGK1 expression	siRNA-mediated silencing of SGK1 inhibited the acute social isolation	Kaji and Nonogaki, 2010

*SGK1, Serum- and Glucocorticoid-regulated Kinase 1; GR Glucocorticoids Receptor; Oct1, Octamer-binding protein 1; SGK3, Serum- and Glucocorticoid-regulated Kinase 3; NDRG1, N-Myc Downstream-Regulated Gene 1; Dsg1, Desmoglein 1; UCMS, Unpredictable Chronic Mild Stress; SNI, Spared Nerve Injury; CUS, Chronic Unpredictable Stress; SGZ, Subgranular Zone; DG, Dentate Gyrus; PAG, Periaqueductal Gray; NSCs, Neural Stem Cells.*

## SGK1, Neurodevelopment and Neurotrophic Factors

Decreased hippocampal volumes have been found in depressed human subjects exposed to chronic stress (Campbell and MacQueen, 2006; Schmidt et al., 2011). This finding supports the hypothesis that chronic stress can inhibit neurogenesis and retract dendritic processes, resulting in neuronal loss in the hippocampus (Schmidt and Duman, 2007). Neurotrophic factors (or neurotrophins) are key determinants in supporting neuronal survival and differentiation, playing an essential role in the process of regulating neuronal formation in neural networks (Meng et al., 2019).

Among neurotrophins, brain-derived neurotrophic factor (BDNF) has a leading role in the pathophysiology of depression (Numakawa et al., 2014; Zhang et al., 2017). Indeed, chronic stress has been described to decrease the BDNF blood level among MDD patients (Molendijk et al., 2011; Kreinin et al., 2015) as well as its expression in the hippocampal dentate gyrus of post-mortem brains of MDD subjects (Castrén and Rantamäki, 2010). Recently, BDNF was suggested to have a significance as predictive biomarker for the treatment of mood disorders (Polyakova et al., 2015). It should also be mentioned that BDNF and VEGF could play a synergistic role in the complex of mood disorders. VEGF is a multifunctional growth factor that stimulates not only angiogenesis, but also neurogenesis with neuroprotective properties (Carmeliet and De Almodovar, 2013).

Brain-derived neurotrophic factor is a neurotrophin highly expressed in the brain, especially in the hippocampus and cortical region, with a key role in the maintenance of neurons in the CNS. BDNF exerts its functions binding with high affinity the receptor TRKB, which activates several downstream intracellular signaling pathways, leading to neuronal survival, synaptic plasticity, and neurogenesis (Numakawa et al., 2013). Since both BDNF/TRKB and GCs/GR systems are involved in neurogenesis, functional crosstalk between them has been a widely studied research topic. SGK1 presumably participates in the signaling of BDNF, which stimulates the PI3-kinase and thus probably SGK1. Since BDNF influences neuronal survival, mood, and long-lasting memory formation and intensifies synaptic plasticity with neuro-regenerative effects by weakening the processes of neuronal degeneration, SGK1 may contribute to several BDNF-dependent neuropsychiatric disorders including depression (Lang et al., 2010). However, the molecular mechanisms by which SGK1 could be a convergence point between glucocorticoids and BDNF in neurogenesis are still not completely clear. Current knowledge indicates that GCs/GR system appears to affect the intracellular signaling pathways involved in the BDNF/TRKB system (Numakawa et al., 2013). In rat cultured cortical neurons, the treatment with dexamethasone (DEX), a synthetic glucocorticoid, decreases the indirect association between TRKB and SHP2 (Src homology-2 domain-containing phosphatase 2), negatively modulating the ERK-mediated expression of synaptic proteins such as NR2A and synapsin I (Kumamaru et al., 2011). Furthermore, the chronic exposure to DEX or corticosterone, a natural glucocorticoid, inhibits the direct binding TRKB/GR due to the decline in GR expression. Thus, the lack in the interaction of the two receptors leads to the suppression of BDNF-induced glutamate release by impairing the activation of the phospholipase C-gamma (PLCɤ)/Ca^2+^ system in the above mentioned cellular model (Numakawa et al., 2009). Indeed, in the mouse hippocampal BZ cell line, GR is able to repress the transcription of *BDNF* gene, through binding an unidentified transcription factor at the gene promoter region (Chen et al., 2017).

Although the mechanisms underlying the pathophysiology of MDD are still not well elucidated, growing evidences converges to the neurotrophic hypothesis of depression (Duman and Li, 2012; Jaggar et al., 2019). In this context, BDNF and vascular endothelial growth factor (VEGF) reduced levels are closely linked with neuronal atrophy in some brain regions implicated in MDD by affecting the hippocampal volume and vascularization, and inducing cognitive decline (Duman and Monteggia, 2006; Duman et al., 2016). In particular, decreased levels of the neurotrophins and their respective receptors TRKB and FLK1, have been reported in the PFC and hippocampus in post-mortem studies of subjects with depression (*n* = 10 vs. *n* = 24 controls; *n* = 14 vs. *n* = 14 controls) (Karege et al., 2005; Qi et al., 2015) as well as in rodent models of chronic stress (Heine et al., 2005; Elfving et al., 2010; Howell et al., 2011). The synergistic actions of BDNF and VEGF on neurogenesis and their antidepressant-like effect is corroborated by other evidence, demonstrating that BDNF stimulates both expression and release of VEGF in neuroblastoma cells and rat primary cortical neurons (Deyama et al., 2019). Moreover, the BDNF-mediated induction of cortical neurons dendrite complexity is blocked by a selective VEGF–fetal liver kinase 1 receptor antagonist, indicating that BDNF neurotrophic and antidepressant-like actions require the downstream VEGF signaling (Deyama et al., 2019). The molecular mechanism according to which SGK1 may modulate VEGF levels has been elucidated through a viable *SGK1* knockout mouse model. The ablation of *SGK1* resulted in a robust decrease in phosphorylation of the target protein NDRG1 accompanied by down-regulation of two NF-κB inhibitory components: IκBα and NF-κB2/p100. The resulting enhancement of NF-κB signaling increased the expression of the downstream target protein, VEGF-A, thus disrupting the normal development and vessel formation (Zarrinpashneh et al., 2013). Based on these findings, SGK1 appears to exert, under chronic stress condition, its anti-neurogenic activity by negatively regulating the expression of both BDNF and VEGF neurotrophins.

## SGK1, Neurodevelopment and Cytokines

The role of neuro-inflammation in depression and consequently, the role of SGK1 in this specific phenotype is, in some ways, controversial. The inflammation resulting from physical stress has shown, at least in rats, to be associated with depression in presence of elevated levels of SGK1, both in blood and in hippocampal and amygdala neurons, within the framework of glucocorticoid-dependent depressive effects (Anacker et al., 2013b; Girgenti and Duman, 2018). Vice versa, the depression resulting from post-traumatic stress such as childhood and adolescent maltreatments is associated with a strong inflammatory component in the presence of decreased levels of SGK1, accompanied by reduced hippocampal neurogenesis (Pariante, 2017). Thus, it emerges that different depressive forms, although clinically part of the major depressive disorder, may have different molecular characterizations depending on the origin of the damage. The difference may be also the result of different cellular components involved. In depression with post-traumatic inflammation, it mainly involved the glial component (microglia and astrocytes), which plays a putative role of innate immunity in CNS (Inoue et al., 2016). Moreover, a novel line of research points to a possible role of SGK1 in mediating Th17-dependent inflammatory effects, in particular diets and stress conditions (Kleinewietfeld et al., 2013; Wu C. et al., 2013; Hernandez et al., 2015; Spagnuolo et al., 2018). In fact, high sodium chloride diets may drive inflammation and autoimmune disease by the induction of pathogenic Th17 cells through the expression of SGK1. It appears evident in this light that a reasonable identification of SGK1 as a pharmacological target requires a careful evaluation of the molecular pathways and cellular actors that are involved. Collectively, the causative role of SGK in MDD is made more confusing in presence of such large number of factors including altered distribution in distinct cell types, different levels of expression and protein interaction, and co-participation of different splicing variants (Lang et al., 2010). As the roles of SGKs may be dramatically divergent among different types of cells in brain, analysis of SGKs and its interactome in each type of cells is required.

## SGK1, Target of New Antidepressant-Mimicking Compounds

The World Health Organization (WHO) estimates that around 350 million individuals suffer from depressive disorders worldwide and up to 40% of patients do not adequately respond to antidepressant medications (James et al., 2018). Over the past 30 years, the understanding of depression was mainly based on the monoamine-deficiency hypothesis, which identifies an association between the occurrence of depression and deficiencies of three major monoamine transmitters as 5-hydroxytryptamine (5-HT), norepinephrine (NE), and dopamine (DA). Therefore, the most commonly employed pharmacological treatments of MDD consist of monoamine oxidase inhibitors, tricyclic antidepressants (TCAs), selective serotonin reuptake inhibitors (SSRIs), and serotonin/noradrenaline reuptake inhibitors (SNRIs), all triggering augmentation of synaptic monoamine levels (Otte et al., 2016). However, these antidepressant treatment have an efficiency of only 60 to 65% with a remission rate of ∼30% (Montes et al., 2004; Block and Nemeroff, 2014), and often require 14 to 21 days or longer for the onset of antidepressant efficacy. The elucidation of new molecular mechanisms in the pathogenesis of the MDD has accelerated the effort to develop novel and effective antidepressants.

In recent years, accumulating evidence has enforced the idea that medicinal herbs and constituents isolated from plant extracts possess psychotherapeutic activities in the treatment of psychiatric disorders, including depression (Zhang, 2004). Baicalin, a major active flavonoid compound extracted and purified from the dry roots of *Scutellaria baicalensis*, has been described to hold several biological properties, including antioxidant, anti-inflammatory, and neuro-protective actions (Zhou et al., 2015a; Shi et al., 2017). Interestingly, baicalin has been reported to overcome the brain-blood barrier (Liu et al., 2007) and exhibited antidepressant effects (Xiong et al., 2011). In a mouse model of depression, obtained by repeated exogenous corticosterone (CORT) injections, a 21-day treatment with baicalin (10 and 20 mg/kg) reversed CORT injection-induced depressive-like behaviors and restored serum corticosterone levels. Indeed, baicalin enhanced the mRNA and protein expression of glucocorticoid receptor (GR) and BDNF, whereas down-regulated SGK1 expression in the hippocampus (Li et al., 2015). Accordingly, in the same mouse model of depression, baicalin treatment (40, 80, or 160 mg/kg for 4 weeks) was reported to restore chronic CORT-induced suppression of hippocampal neurogenesis. In particular, baicalin restored the chronic CORT-induced decrease in GR protein levels, the rate in GR nuclear translocation, as well as the intensification of GR phosphorylation at Ser203 and Ser211. In addition, baicalin treatment also normalized the chronic CORT-induced increase of both FKBP5 protein levels and SGK1 phosphorylation at Ser422 and Thr256. Taken together, these findings suggest that baicalin counteracts anxiety/depression-like behaviors, promoting hippocampal neurogenesis through the regulation of SGK1- and FKBP5-dependent GR phosphorylation (Zhang et al., 2016). Although present findings elucidate the molecular mechanisms behind the antidepressant activity of baicalin, nevertheless, further studies are required to clarify the precise correlation between SGK1 and BDNF in neurogenesis. Recently, Icariin, another flavonoid isolated from *Herba Epimedii*, demonstrated to play potential antidepressant-like effects in several depression models (Pan et al., 2006, 2007, 2010). In a rat model of depression induced by unpredictable chronic mild stress (UCMS), the oral administration of icariin (20 and 40 mg/kg) for 35 days attenuated the development of depression-like behaviors. It was noteworthy that, similarly, to baicalin, icariin restored the UCMS-induced increases in the levels of cytosolic GR and SGK1 in both the hippocampus and the prefrontal cortex. Icariin also partially reversed the upregulation of FKBP5 and nuclear localization of GR in the hippocampus and in the prefrontal cortex, respectively (Wei et al., 2016). These data suggest that the antidepressant-like effects of icariin, similarly to the effects exerted by fluoxetine, a SSRI widely used in the psychopharmacology of MDD, can be explained by the restoration of the physiological negative feedback of the HPA axis and the canonical neurogenesis in related brain regions. Interestingly, antidepressant-like effects were also observed in mice model of CORT-induced depression treated with oleanolic acid, a triterpenoid compound present in natural plants. The antidepressant-like effect of the treatment with oleanolic acid (10 and 20 mg/kg for 21 days) appeared to be mediated by the down-regulation of the SGK1 and GR expression concomitantly to the upregulation of the hippocampal BDNF-AKT/mTOR signaling pathway (Dong et al., 2019). A recent *in vitro* study supported the hypothesis that SGK1 could play a key role in mediating the antidepressant effects of different natural compounds. In this study, leonurine, also called SCM-198 (4-guanidino-n-butyl syringate), a chemically synthesized compound based on a bioactive alkaloid extracted from the herbaceous perennial plant *Leonurus cardiaca*, has been demonstrated to increase cell viability of corticosterone-induced PC12 cells, with the maximal pro-survival effect at 60 μM. Indeed, the treatment with lenourine increased cell area, total neurite length, and maximum neurite length by inducing the expression of GR, BDNF, NT-3, and BCL-2, and inhibiting the SGK1 expression. Notably, the protective effect of leonurine against CORT-induced cell death, as well as its ability to increase the expression of the above-mentioned genes was potentiated by SGK1 inhibition through GSK650394 (20 μM) pre-treatment. These data suggest that leonurine may exert antidepressant effects, modulating the neurite outgrowth and neurotrophic activity as observed in CORT-cultured PC12 cells, and that this effect may depend on the negative modulation of GR/SGK1 signaling (Meng et al., 2019). In a different study, the ethanolic extract from the *Dipterocarpus alatus* leaf, containing flavonoids (luteolin-7-*O*-glucoside, kaempferol-3-glucoside, rutin) and phenolic acids (gallic acid, ferulic acid, and caffeic acid) as major constituents, showed an antidepressant activity, resulting in the attenuation of anhedonia (increased sucrose preference) and behavioral despair (decreased immobility time in tail suspension test and forced swimming test) in an UCMS mouse model of depression. Administration of the extract (100 and 500 mg/kg for 3 weeks) not only decreased the UCMS-induced elevation of serum CORT levels and the hyperactivation of the HPA axis, but also normalized, in a dose-dependent manner, the mRNA expression of SGK1, cyclic AMP-responsive element binding (CREB) and its downstream target BDNF in the frontal cortex and hippocampus, effects similar to those observed with imipramine (20 mg/kg) (Daodee et al., 2019). In addition, in an *in vitro* assay, the extract exerted also a partial selective inhibition on the enzyme monoamine oxidase (MAO)-A, whose abnormal activity is reported to play an important role in depressive disorders (Naoi et al., 2018). Although these findings are relevant for understanding the antidepressant mechanism of *Dipterocarpus alatus* leaf extract, further studies are necessary to clarify the specific mechanisms involved in the neurogenesis in dentate gyrus and in other signaling pathways involved in regulation of HPA axis.

Gypenoside, a very active saponin isolated from *Gynostemma pentaphyllum*, is known to have a variety of pharmacological properties, including anti-inflammatory (Liou et al., 2010), antioxidative (Schild et al., 2012), neuroprotective (Choi et al., 2010), and antidepressant-like activities (Mu et al., 2016). Gypenoside administration (100 mg/kg for 3 weeks) in chronic CORT-induced depressed mice significantly reverted the stress-dependent increase of hippocampal SGK1 protein levels as well as the decreased activation of CREB-BDNF signaling, suggesting that the antidepressant-like effects of gypenosides are related to both the inhibition of SGK1 expression and the neurogenesis stimulation via GR-dependent pathway (Wu J. et al., 2013; Yi et al., 2018).

Recently, Moore A. et al. reviewed a fair amount of evidence showing that resveratrol, a polyphenol with antioxidant and anti-inflammatory properties that highly present in grape skins and therefore in red wine, abrogates depressive-like behavior and neuroinflammatory response, and enhances hippocampal neurogenesis in several animal model of depression. The neuroprotective effects exerted by resveratrol administration (10–80 mg/Kg/die) mainly arise from the enhancing of CREB/BDNF signaling as well as the regulation of HPA axis function (Moore et al., 2018). In addition, resveratrol has also been shown to have an *in vitro* inhibitory activity on SGK1 in HUH7 human hepatoma cells (Catalogna et al., 2019). The antidepressant effects of resveratrol are also being studied in humans, through a double-blind randomized clinical trial on 60 patients with depression, already in phase four of the experimentation U. S. Nation Library Of Medicine and ClinicalTrials.gov (2020). Although more studies elucidating the precise molecular mechanism are needed, taken together these finding suggest that the effects of resveratrol on the CNS are probably mediated, at least in part, by its action on SGK1.

**FIGURE 1:**
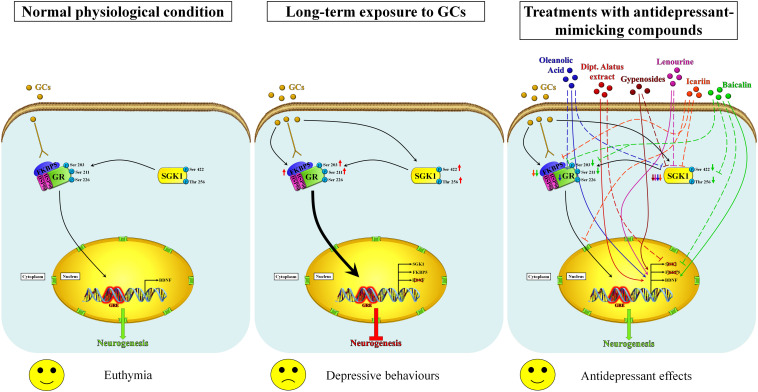
Proposed mechanisms of action of nutraceuticals in the restoration of neurogenesis by modulating the GR/SGK1 signaling pathway. In the normal physiological state, the exposure of neuronal cells to basal levels of glucocorticoids activates GR and promotes its translocation into the nucleus, regulating thus neurogenesis **(left panel)**. Prolonged exposure to high concentrations of glucocorticoids increases the expression of FKBP5, which impairs cytosolic GR binding capability and therefore the ultra-short negative feedback loop on GR sensitivity, increasing thus the negative effects of glucocorticoids. Furthermore, high glucocorticoids levels also improve SGK1 activity by increasing the phosphorylation of SGK1 at Ser422 and Thr256, which in turn causes the phosphorylation of GR at Ser203 and Ser211. The hyperphosphorylation of GR stimulates its nuclear translocation (thick arrow) and, by binding unidentified co-repressor factors, down-regulates the BDNF expression that results in impaired neurogenesis and then in depressive-like behaviors **(middle panel)**. Nutraceuticals may counteract the effects of long-term exposure to glucocorticoids by restoring normal GR/SGK1 signaling through similar molecular mechanisms that lead to improved neurogenesis and antidepressant effects **(right panel)**. Inhibitory and enhancing actions are indicated by dashed lines and arrows, respectively. Baicalin (green) is able to counteract the GCs-induced effects by enhancing the BDNF expression, downregulating the expression of SGK1, decreasing the FKBP5 protein levels as well as the phosphorylation rate of both GR and SGK1. Icariin (orange) decreases the SGK1 and FKBP5 protein levels and affects the GR nuclear translocation. Lenourine (violet) stimulates the BDNF expression whereas negatively regulates the SGK1 expression. Gypenosides (brown) reduces the SGK1 protein levels and enhances the BDNF expression. Dipt. Alatus extract (red) normalizes the mRNA levels of SGK1 and stimulates the expression of BDNF. Oleanolic acid (blue) down-regulates the expression of both SGK1 and GR as well as up-regulates the expression of BDNF.

Considering that oxidative stress is unequivocally associated with the advancement of depression, all this evidence suggests that nutraceuticals may help to reduce the symptoms of depression, notably via dietary supplementation with the minimization of depression risk due to their important anti-inflammatory and antioxidative natures. All these studies highlighted that the antidepressant effects of antioxidative compounds are related to the normalization of the “stress protein” SGK1 level in parallel with the restoration of neurogenesis (Figure 1); for this purpose, advanced investigations are needed to fully understand the mechanism of actions including neuroprotection, biotransformation of their metabolites in the body, and potential interactions with molecular target involved in the depression. Nowadays, except for resveratrol, the described studies on the above-mentioned nutraceuticals are still at a very early stage and further confirmations on their antidepressant effects in specific clinical trials are required. More recently, several studies focused on the role of ketamine and its derivatives in the treatment of treatment-resistant forms of depression (TRD) (Bratsos and Saleh, 2019; Park et al., 2019; Kryst et al., 2020). Under these specific conditions, the trend of SGK1 appears paradoxical with respect to that observed in the classic stress-dependent forms of depression (Ficek et al., 2016; Qiao et al., 2019). The explanations for this paradoxical up-regulation of SGK1 in response to the ketamine treatment could lie in at least two key points: first, the neuronal model of glutamatergic activation differs from the canonical assessments of dopaminergic and serotoninergic neurons target for classical anti-depressants; second, it should be borne in mind that the rapid effect of ketamine is accompanied by a controlled dissociative state that could be related to high levels of anxiety-dependent stress probably with increase glucocorticoid release. It is interesting to note that in response to the treatment with ketamine and to the ketamine-dependent SGK1 activation, a deep modulation in the FOXO activity (Qiao et al., 2019) and presumably in its transcriptional functionality has been also noticed, which could, in turn, explain the rapidity of the antidepressant effects of ketamine. However, further studies will be necessary to understand the potential role of SGK1 as a pharmacological target for the ketamine and its derivatives in depressive conditions refractory to traditional therapies.

## Conclusion

**FIGURE 2:**
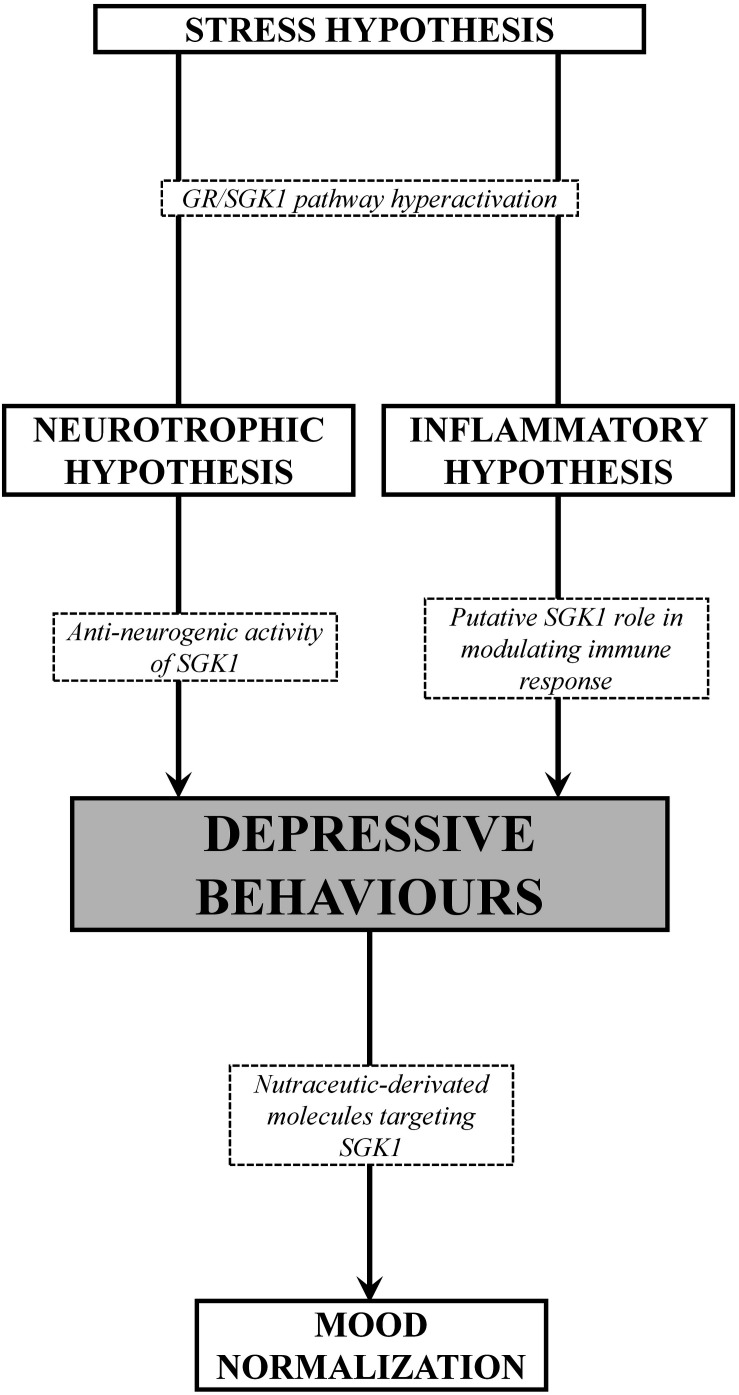
Schematic overview of the SGK1 role in different pathogenetic hypothesis of MDD. Under stress conditions, the increase of glucocorticoids induce a dysregulation of the GR/SGK1 axis, thus resulting in depressive behaviors, presumably dependent on anti-neurogenic activity exerting a negative regulation in the expression of neurotrophins, and/or the immune/inflammatory response as well. On the other hand, nutraceuticals-derived molecules with antioxidant, anti-inflammatory and neuro-protective properties, exhibit antidepressant effects due, at least in part, to the negative modulation of SGK1.

In the light of the above reviewed data, it is therefore clear that several hypotheses may be taken into account in the explanation of the pathogenesis of depression. At present, it appears more appropriate to refer to a depressive spectrum disease than to an organically structured depressive disease. Thus, for a correct clinical and molecular approach, it is necessary to first evaluate and subclassify the causes and symptomatic forms of the specific depressive disorder, and later to approach the more balanced therapeutic methodology, congruent with the most plausible pathophysiological hypothesis. The more resistant forms of MDD, which are refractory to traditional treatments, could offer a first experimentation frontier for new synthetic or nutraceutic-derivated molecules targeting SGK1. In fact, SGK1 could play as a neuronal resensitization factor and in any case a convergence point of different and often opposite signal pathways. In this light, by taking in account genetic heterogeneity and several environmental factors, it may be possible tailor therapeutical approaches to be more individualized, effective, and better tolerated. From a genetic point of view, the SGK1 kinase appears to be a clear piece for a unifying vision of the overall pathological forms examined (Figure 2). The role of gene mutations or functional dysregulations of SGK1 needs to be investigated in a well-designed clinical cohort of patients in order to ultimately define the role of SGK1 in the pathogenetic evolution of this pathological spectra. However, it is evident from what has been said so far that SGK1 is proposed as a therapeutic molecular target for a modern therapy of the depressive disorder and that efforts in this sense will increasingly clarify how much this new candidate gene can provide the explanations missing in the univocal vision of the disease.

## Author Contributions

VD and RA conducted the literature search, wrote the manuscript and prepared the tables and figures. NP and MG reviewed and edited drafts of the manuscript. All authors contributed to reading and approving the final version of the manuscript.

## Conflict of Interest

The authors declare that the research was conducted in the absence of any commercial or financial relationships that could be construed as a potential conflict of interest.
